# Development of near-infrared spectroscopy (NIRS) for estimating organic matter, total carbon, and total nitrogen in agricultural soil

**DOI:** 10.1016/j.mex.2024.102798

**Published:** 2024-06-15

**Authors:** Natchanon Santasup, Parichat Theanjumpol, Choochard Santasup, Sila Kittiwachana, Nipon Mawan, Lalicha Prantong, Nuttapon Khongdee

**Affiliations:** a Department of Plant and Soil Science, Faculty of Agriculture, Chiang Mai University, Chiang Mai 50200, Thailand; b Postharvest Technology Research Center, Faculty of Agriculture, Chiang Mai University, Chiang Mai 50200, Thailand; c Department of Chemistry, Faculty of Science, Chiang Mai University, Chiang Mai 50200, Thailand; d Department of Highland Agriculture and Natural Resources, Faculty of Agriculture, Chiang Mai University, Chiang Mai 50200, Thailand

**Keywords:** Chemometrics, Prediction, Model, Highland and lowland soil properties, Non-destructive organic matter, total carbon, and total nitrogen measurement in agricultural soil

## Abstract

The analysis of soil organic matter (OM), total carbon (TC), and total nitrogen (TN) using traditional methods is quite time-consuming and involves the use of hazardous chemical reagents. Absorbance spectroscopy, especially near-infrared (NIR), is becoming more popular for soil analysis. This method requires little sample preparation, no chemicals, and a single spectral analysis to evaluate soil properties. Thus, this research aimed to develop an NIR spectroscopy method for the analysis of OM, TC, and TN in agricultural soils. These findings can provide a good concept of using PLS regression with NIR techniques. The method is as follows:

• Topsoil (0–20 cm) samples were collected from various agricultural fields. OM, TC, and TN were analyzed using traditional methods and NIR spectroscopy.
• NIR spectra were obtained using an FT-NIR spectrometer, original spectral including with Savitzky–Golay smoothing, standard normal variate (SNV) and multiplicative scatter correction (MSC) preprocessing method were used to create a predicted model through Partial Least Squares (PLS) regression with 65 % calibration, and the rest 35 % for validation.
• The results showed significant relationships between measured soil properties (SOM and TC) and NIR absorbance spectra in agricultural soil (*R*^2^ of calibration and validation higher than 0.80).

## Graphical abstract

**Figure:**
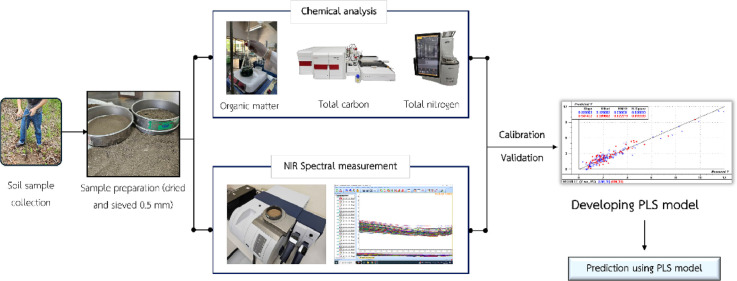

Specifications table

**Table:** 

Subject area:	Agricultural and Biological Sciences Agricultural and Biological Sciences
More specific subject area:	Organic matter, total carbon, and total nitrogen analysis in soil
Name of your method:	Non-destructive organic matter, total carbon, and total nitrogen measurement in agricultural soil
Name and reference of original method:	Wet oxidation methods [1] Carbon combustion methods Nitrogen combustion methods
Resource availability:	Data will be made available on request.

## Background

Soil organic matter (SOM) is an essential indicator of good soil quality. It is closely associated with soil structural stability, aggregation, infiltration, nutrient cycling, fertility, and soil erodibility [2]. Besides that, carbon in the soil also has a crucial role in mitigating climate change because carbon dioxide (CO_2_) can be removed from the atmosphere and stored in the soil [3]. Furthermore, nitrogen in soil is a crucial element for plant structure. It participates in creating proteins, nucleic acids, chlorophyll, and enzymes and it's essential for photosynthesis in plants [4]. Monitoring the soil status is in great demand in precision agriculture to adjust practices such as tillage, fertilization, and irrigation. Understanding soil characteristics can enhance farmers, enabling them to make well-informed decisions about their agricultural practices. This knowledge significantly improves the efficiency of operations, practices, and treatments applied in soil management [5]. However, standard analytical procedures like wet chemistry requires various chemical reagents, laborious, and extremely time-consuming, especially when dealing with a high spatial sampling density [6]. In recent years, proximal soil sensing techniques, particularly NIR spectroscopy, have taken a greater significance in soil science, overcoming the drawbacks of conventional laboratory methods. Near-infrared spectroscopy (NIRS) is a technique that observes the interaction between electromagnetic waves in the 800–2500 nm region and samples, spectral data collected are overtone oscillations and combinations of organic compounds in the produced C—H, O—H, S—H, N—H, and C=O bonds [7]. Spectrals were then applied with the chemometrics process consisting of a preprocessing method and multivariate calibration. This process aimed to establish a correlation between two matrices; the predictor variables X (soil spectral data) and the variables to be predicted Y (soil chemical properties) [8]. However, the accuracy of predictions varied due to the regions, soil pedological characteristics, and particularly site-specific practices.

Therefore, our idea was to develop an appropriate NIR technique to evaluate organic matter, total carbon, and total nitrogen in agricultural soil. The output of this study can improve the precision of determining soil fertility and fitting fertilizer levels for crop production. This research approach aims to overcome the drawbacks of conventional laboratory methods by offering a cost-effective, non-destructive, environmentally friendly, repeatable, and reproducible analytical technique. Even though NIR technique is now able to provide real-time data [9], but farmers in many regions have not yet widely adopted the real-time NIR technique. Additionally, soil analysis services continue to primarily use traditional methods. By switching to NIR technology, we can save analysis time and analyze more soil samples. Moreover, it is free from hazardous substances while ensuring the accuracy and reliability of the analytical results produced.

## Method details

**Fig. 1:**
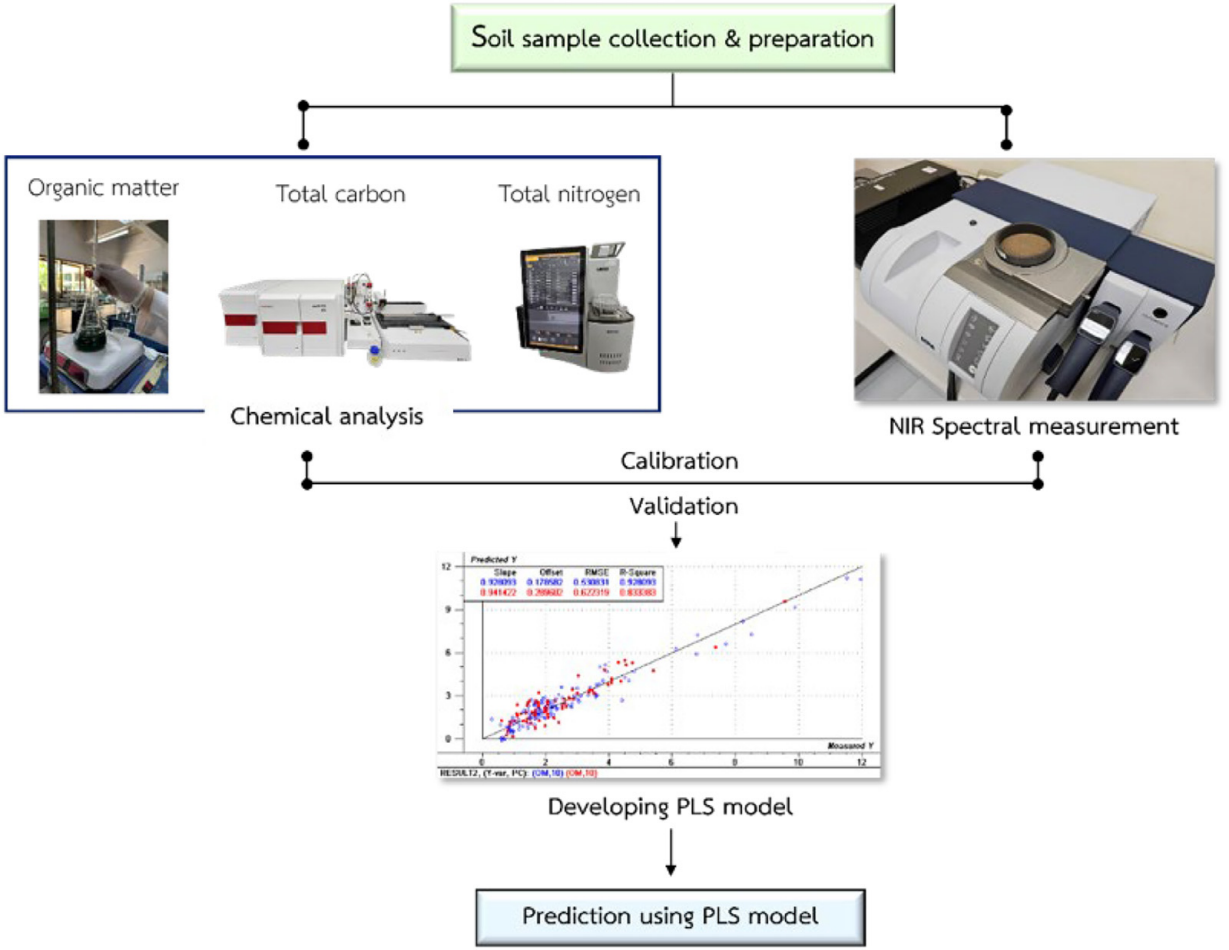
A diagram of the near-infrared spectroscopy (NIRS) technique procedure.

Developing organic matter, total carbon, and total nitrogen measurement using near-infrared spectroscopy (NIRS) technique as illustrated in Fig. 1.

### Soil sample collection and chemical analysis

A total of 200 topsoil (0–20 cm) samples were collected by a composite method using an auger from various agricultural fields in Chiang Mai Province, Thailand. After arrival at the laboratory, the grass and plant debris on the surface were removed. The soil samples were naturally air-dried and passed through a 0.5 mm mesh sieve before chemical analysis. The Walkley and Black chromic acid wet oxidation method was used for organic matter analysis. This involved oxidizing organic carbon in soil with 0.167 M potassium dichromate (K_2_Cr_2_O_7_) solution in concentrated sulfuric acid. Then, measure the remaining unreduced dichromate by back-titrating with ferrous sulfate using the o-phenanthroline-ferrous complex as an indicator. The total carbon percentage was evaluated by putting 0.05 g of soil samples into a C-free boat and placing them in a C-S Analyzer (analytikjena). Total nitrogen was analyzed: about 0.10–0.50 g soil samples were encapsulated within N-free tin foil and placed in the N-Analyzer (LECO). The total concentration of nitrogen was then expressed as a percentage. The average range of organic matter, total carbon, and total nitrogen in the soil samples utilized for this investigation is presented in Table 1.

**Table 1:** Range of soil organic matter, total carbon and total nitrogen in collected agricultural soils.

Soil Chemical Constituent	Range	Average	S.D.*
Soil organic matter (%)	0.30–11.96	2.47	1.84
Total carbon (%)	0.46–6.21	1.66	0.95
Total nitrogen (%)	0.02–0.47	0.17	0.10

⁎ *Noted*: S.D. = Standard Deviation.

### NIR spectral data acquisition and preprocessing

The soil samples were placed in a rotating cup with a 100 mm of diameter. Near-infrared (NIR) spectral data were collected using an FT-NIR spectrometer (BRUKER OPTIK GmbH, Germany) across the 4000 to 12,500 cm^− 1^ range (800 to 2500 nm), the spectrometer executed 32 scans with a 16 cm^−1^ resolution. Background measurements entailed using the FT-NIR spectrometer to gauge the internal gold-coated diffuse reflector within the integrating sphere, which was repeated hourly. The Opus software facilitated gathering spectral data, with subsequent exportation of FT-NIR spectra to the Unscrambler software for chemometrics analysis.

### Method calibration

In order to determine the most accurate model for predicting the concentration of organic matter, total carbon, and total nitrogen in the soil, we employed raw spectral data along with three preprocessing methods: Savitzky–Golay smoothing, standard normal variate (SNV), and multiplicative scatter correction (MSC). The prediction models were developed using partial least squares (PLS) regression, with 65 % of soil samples employed for calibration and the remaining 35 % for validation. The performance of the prediction was assessed using the coefficient of determination (*R^2^*) and the root mean square (RMSE). The highest *R^2^* and the lowest RMSE were considered the best prediction model.

## Method validation

**Fig. 2:**
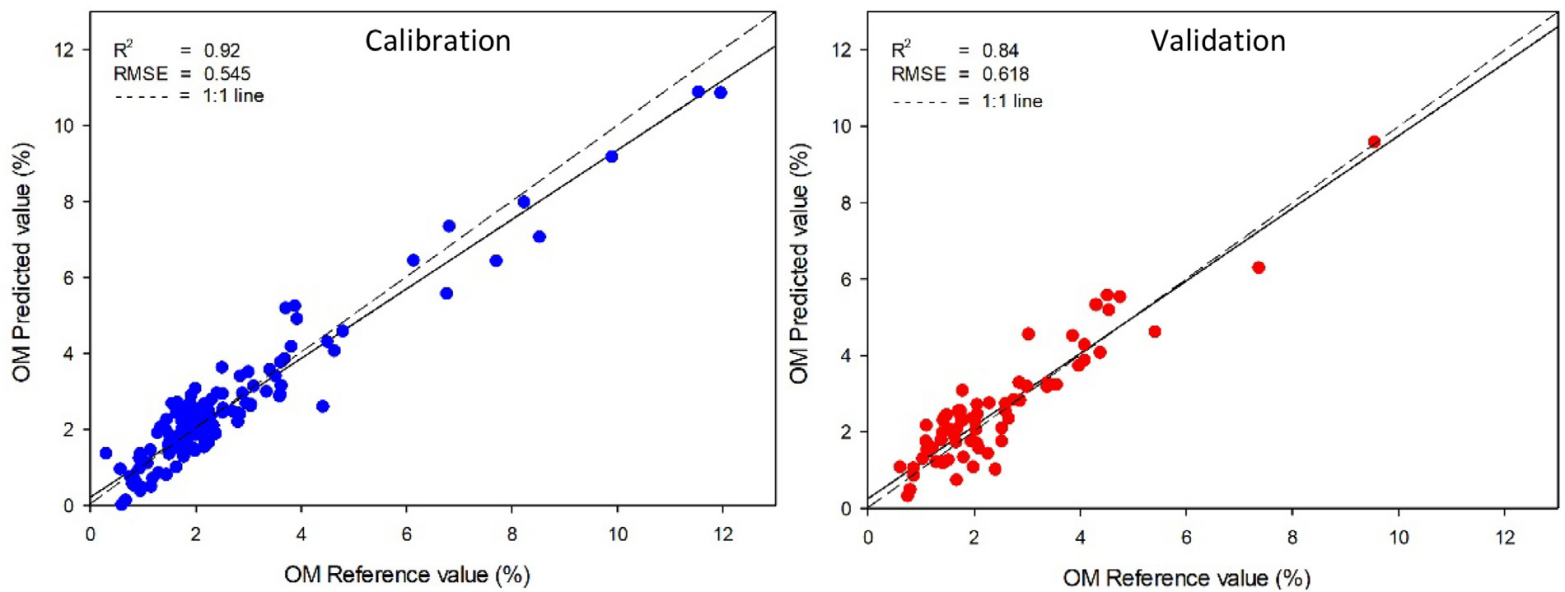
Comparisons of soil organic matter, measured by wet oxidation and predicted by NIR spectroscopy using smoothing preprocessing method with PLS model.

**Fig. 3:**
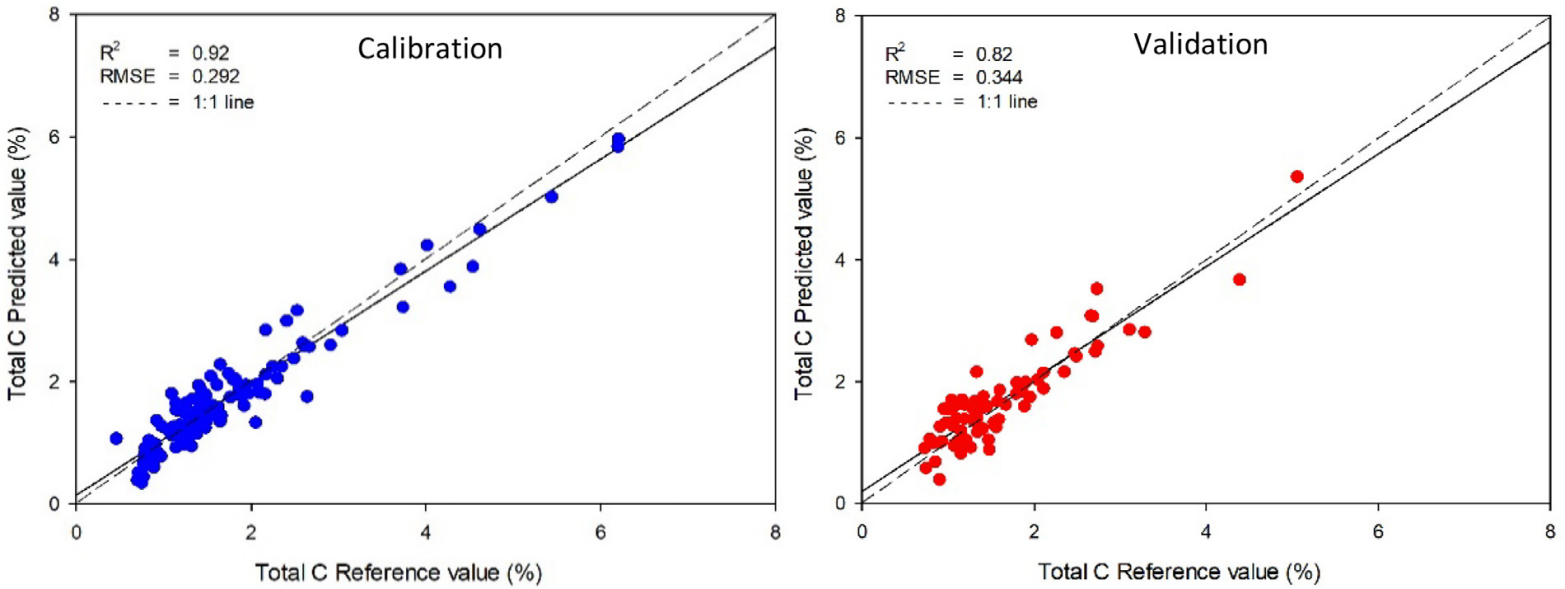
Comparisons of total carbon, measured by combustion and predicted by NIR spectroscopy using smoothing preprocessing method with PLS model.

**Fig. 4:**
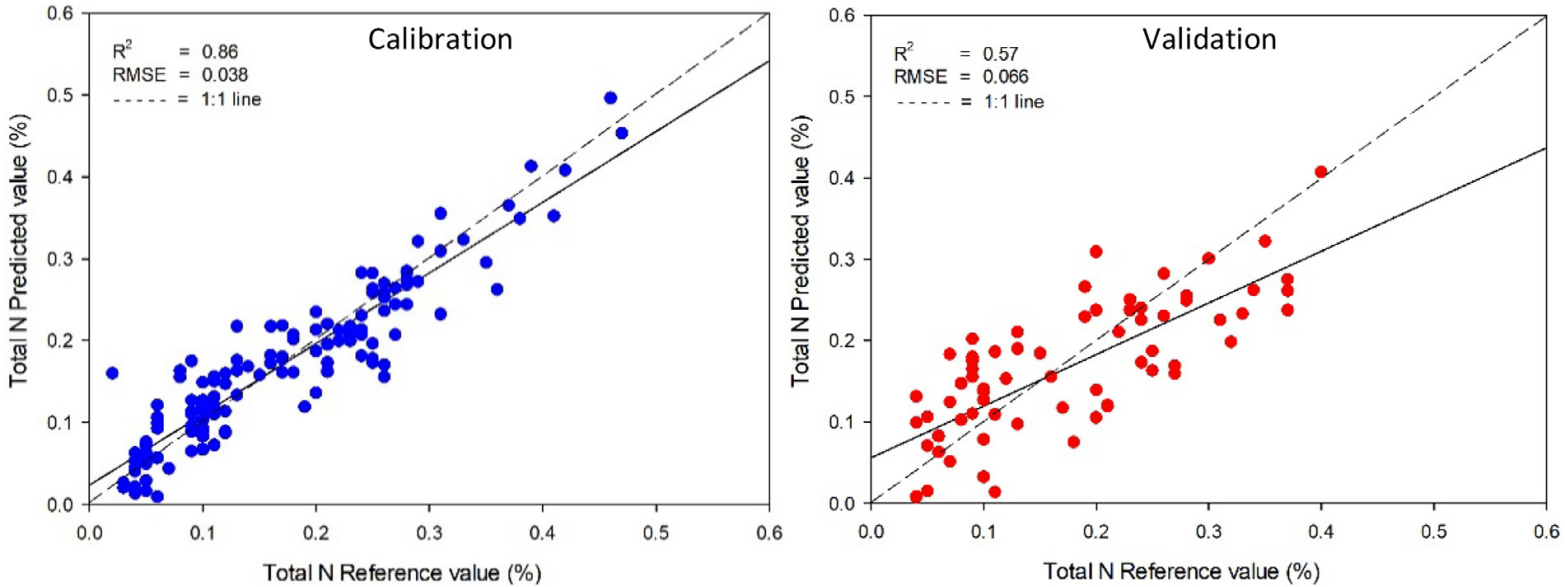
Comparisons of total nitrogen, measured by combustion and predicted by NIR spectroscopy using smoothing preprocessing method with PLS model.

The calibration and validation statistics of prediction models created by PLS were presented in Table 2. These models were created to establish a relationship between the absorbance of near-infrared spectroscopy and the reference values of organic matter, total carbon, and total nitrogen in soil obtained using standard analysis methods. The best-predicted model for organic matter was obtained from smoothing pre-processing with PLS due to the highest *R^2^* (0.84) and the lowest RMSE (0.618 %) of validation samples (Fig. 2). For total carbon, smoothing pre-processing cooperated with PLSR and also created the most accurate model with the highest *R^2^* (0.82) and the lowest RMSE (0.344 %) of validation samples (Fig. 3). While the predicted model of total nitrogen in calibration sets met the requirements, with *R^2^* and RMSE values ranging from 0.78–0.89 to 0.067–0.075 % respectively, the model was unable to predict total nitrogen in the soil accurately. This is evident from the low values of *R^2^*, which ranged from 0.44 to 0.57. The most accurate model, obtained through smoothing pre-processing with PLS, was shown in Fig. 4 and the comparison result of this study with previous studies was shown in Table 3. The results indicated that the model developed using PLS with NIR techniques could be an alternative method to predict organic matter and total carbon concentrations in agricultural soil.

**Table 2:** Model parameters and statistical indices for prediction of SOM using PLS regression with different data preprocessing (800–2500 nm).

Parameter	Spectral pre-processing	Calibration		Validation	
		*R^2^*	RMSE (%)	*R^2^*	RMSE (%)
SOM	Raw spectral	0.93	0.531	0.83	0.622
	**Smoothing**	**0.92**	**0.545**	**0.84**	**0.618**
	SNV	0.81	0.867	0.58	0.992
	MSC	0.84	0.776	0.66	0.895
Total carbon	Raw spectral	0.93	0.274	0.82	0.345
	**Smoothing**	**0.92**	**0.292**	**0.82**	**0.344**
	SNV	0.81	0.446	0.59	0.516
	MSC	0.84	0.398	0.65	0.474
Total nitrogen	Raw spectral	0.89	0.035	0.56	0.067
	**Smoothing**	**0.86**	**0.038**	**0.57**	**0.066**
	SNV	0.82	0.044	0.44	0.075
	MSC	0.78	0.049	0.50	0.071

**Table 3:** The comparison of the best model obtained from this study with previous studies.

Soil parameter	Pre-processing methods	Calibration models	Validation		Comparing references
			*R^2^*	RMSE (%)	
**SOM**	**Smoothing**	**PLSR**	**0.84**	**0.62**	**This study**
SOM	SNV*	SVM*	0.70	0.44	[10]
SOM	1 derivative +smoothing	RF*	0.70	0.62	[11]
SOM	Raw spectral	PLSR	0.77	0.23	[12]
**Total carbon**	**Smoothing**	**PLSR**	**0.82**	**0.34**	**This study**
Total carbon	SNV	Cubist	0.81	0.41	[13]
Total carbon	Smoothing + SNV	Cubist	0.75	0.45	[14]
Organic carbon	First derivative	PLSR	0.81	0.27	[15]
Organic carbon	First derivative	PLSR	0.81	0.26	[16]
Organic carbon	Raw spectral	PLSR	0.78	0.24	[17]
Organic carbon	Smoothing	Cubist	0.73	0.57	[18]

⁎ *Noted*: SNV, standard normal variate; SVM, support vector machine; RF, random forest.

## Limitations

Based on the findings of our research, it can be inferred that the utilization of the Near-Infrared (NIR) method is suitable for evaluating the quantity of organic matter and total carbon content in agricultural soil. The Savitzky–Golay smoothing through the PLSR model is the most appropriate model for evaluating soil organic matter (SOM) and total carbon. It has a coefficient of determination (*R^2^*) of 0.84 and 0.82, as well as a root mean square error (RMSE) of 0.618 % and 0.344 %. Nevertheless, the applicability of our methods to the determination of total nitrogen is limited. This limitation may be attributed to the unsuitability of our method conditions for accurately predicting total nitrogen in soil using NIR spectroscopy. Further investigation should be conducted on alternative data preprocessing and calibration models.

## CRediT authorship contribution statement

**Natchanon Santasup:** Data curation, Writing – original draft. **Parichat Theanjumpol:** Conceptualization, Supervision. **Choochard Santasup:** Conceptualization, Supervision. **Sila Kittiwachana:** Supervision. **Nipon Mawan:** Conceptualization, Methodology. **Lalicha Prantong:** Data curation. **Nuttapon Khongdee:** Conceptualization, Writing – review & editing.

## Declaration of competing interest

The authors declare that they have no known competing financial interests or personal relationships that could have appeared to influence the work reported in this paper.

## Data Availability

Data will be made available on request.
